# Developmentally regulated effects of severe hemorrhage on cardiovascular homeostasis and the arterial baroreflex control of heart rate

**DOI:** 10.14814/phy2.12440

**Published:** 2015-07-21

**Authors:** Mohamed Samhan, Wei Qi, Francine G Smith

**Affiliations:** Department of Physiology & Pharmacology, The Alberta Children’s Hospital Research Institute for Child and Maternal Health, University of CalgaryCalgary, Alberta, Canada

**Keywords:** Baroreflex, cardiovascular, conscious, neonate, newborn, physiology

## Abstract

The purpose of this study was to investigate in developing animals, the cardiovascular responses to severe hemorrhage at which compensatory mechanisms fail and when blood pressure remains decreased after blood loss. Two groups of conscious lambs (Group I: one to two weeks, *N* = 7; group II: six to seven weeks, *N* = 7) were studied. Mean arterial pressure, systolic and diastolic pressures, and heart rate were measured for 20 min before (Control, C) and for 60 min after a fixed hemorrhage of 30% of blood volume. The arterial baroreflex control of heart rate was assessed before (C), and at 30 and 60 min intervals after hemorrhage. Mean arterial pressure decreased for up to 60 min after hemorrhage in both groups of lambs. In group I, heart rate decreased from 200 ± 29 (C) to 164 ± 24 beat min^−1^ at 30 min then increased to 232 ± 45 beat min^−1^ at 60 min, whereas heart rate remained unaltered in group II. With respect to the arterial baroreflex control of heart rate, by 30 min after hemorrhage in group I, there was a decrease in the heart rate range over which the baroreflex operates (*P1*) from 192 ± 13 (C) to 102 ± 9 beats min^−1^; by 60 min after hemorrhage, there was a decrease in minimum heart rate (*P4*) from 72 ± 10 (C) to 32 ± 25 beats min^−1^. In group II, *P1* decreased to a lesser extent than group I from 134 ± 21 (C) to 82 ± 10 beats min^−1^ at 30 min; minimum heart rate (*P4*) decreased from 40 ± 15 (C) to 24 ± 9 and 20 ± 13 beats min^−1^ at 30 and 60 min, respectively. These results provide the first assessment of the arterial baroreflex control of heart rate following blood loss and new evidence that the cardiovascular responses to severe hemorrhage are developmentally regulated.

## Introduction

Hemorrhage elicits numerous physiological responses aimed at the preservation of arterial pressure and tissue perfusion. Initially, there is a tachycardia along with an increase in peripheral vascular resistance that matches the fall in cardiac output so that arterial pressure is maintained. There is also a redistribution of blood flow to vital organs, such as the brain and heart, at the expense of causing ischemia in other organs including the kidney. As blood loss proceeds, arterial pressure and tissue perfusion are not maintained and hypovolemic shock ensues.

Management of hypovolemic shock is guided by the Adanced Trauma Life Support (ATLS®) which suggests classes based upon vital signs and percentage blood loss and has been widely implemented for several decades (Mutschler et al. 2014). For example, in young healthy subjects, blood loss of ∼750 mL (∼15%) results in little change in arterial pressure while there is a mild increase in heart rate. At a blood loss of 800–1500 mL (∼15–30%), a tachycardia occurs to assist in promoting blood pressure which is usually maintained within normal limits due to cardiovascular, endocrine, and renal compensatory mechanisms being elicited. When blood loss exceeds a critical level (∼30–40% of blood volume, or >1500 mL in a 70 kg subject) the aforementioned homeostatic or compensatory mechanisms begin to fail. Arterial pressure declines, sometimes precipitously, which results from a dramatic fall in peripheral vascular resistance accompanied by bradycardia, and a generalized decrease in sympathetic nerve activity (Chien 1967; Schadt and Ludbrook 1991; Koyama et al. 1992). This withdrawal of compensatory responses is defined as hypovolemic shock or decompensated hemorrhage and results in a sustained decrease in blood pressure that does not return to prehemorrhage levels. In shock, blood loss exceeds the body’s ability to compensate and provide adequate tissue perfusion and oxygenation, such failure leading to death in the absence of treatment.

Although responses to severe blood loss early in life are not well characterized, our previous investigations in conscious, chronically instrumented developing sheep have provided some of the first comprehensive reports of the responses to nonhypotensive and moderate hypotensive hemorrhage (or so-called compensated hemorrhage) during postnatal maturation (Smith and Abu-Amarah 1997, 1998; Smith et al. 2000, 2004; Smith 2002). These findings show that hemodynamic, humoral, and renal responses to compensated hemorrhage are considerably different in the newborn period as compared to later in life: For example, after the onset of hemorrhage, there is a dramatic increase in heart rate in newborns but not young adult sheep, while blood pressure is maintained (Smith et al. 2000). Also, mean arterial pressure falls in both age groups, whereas the heart rate response in newborns is more dramatic than that measured in young adult sheep (Smith et al. 2000). We also showed that there is an increase in plasma renin activity at all ages in response to hypotensive blood loss with effects being greater in newborns (Smith et al. 2000). Taken together, then, an increase in heart rate along with activation of the renin–angiotensin system occurs in response to compensated blood loss early in life, assisting the restoration of arterial pressure. To date, however, there have been no investigations in the newborn period into the physiological responses to severe degrees of blood loss such as occurs when the so-called “critical point” is exceeded.

Therefore, the aim of the present study was to investigate the hemodynamic responses to severe hemorrhage at two stages of postnatal maturation (beyond the critical point and at which compensatory mechanisms fail and when blood pressure remains decreased after blood loss). In addition, the arterial baroreflex control of heart rate was assessed before and after blood loss to evaluate the effects of this degree of hemorrhage on the baroreflex.

## Methods

### Animals

Experiments were carried out at least 4 days after the surgery in two separate age groups of conscious chronically instrumented lambs (group I, 12 ± 2 days, *N* = 7, group II, 43 ± 7 days; *N* = 7). Animals were obtained from a local source (Woolfit Acres, olds, AB, Canada) and housed with their mothers in individual pens in the *vivarium* of the Animal Resources Centre of the University of Calgary where they were provided with standard food and water, except during surgery, training, and experiments. All surgical and experimental procedures were carried out in accordance with the “*Guide to the Care and Use of Experimental Animals*” provided by the Canadian Council on Animal Care and with the approval of the Animal Care Committee of the University of Calgary.

### Surgical procedures

Surgery was performed using aseptic techniques as previously described (Smith et al. 1997; Monument and Smith 2003). Briefly, anesthesia was induced with a mask and isoflurane (∼5%) in oxygen, the trachea was intubated, and then anesthesia was maintained by ventilating the lungs with isoflurane (1.5–2.5% in a mixture of nitrous oxide and oxygen (3:2). Under sterile conditions, catheters (Tygon® Microbore Tubing I.D 1.0 mm; O.D. 1.8 mm) were inserted into right and left femoral vessels and advanced to the aorta and inferior vena cava for later pressure measurements, as well as I.V. injections of drugs and solutions. The catheters were tunneled subcutaneously to exit the lamb on the right and left flanks, respectively. Catheters were contained in pouches on a lamb body jacket (Lomir Inc., Montréal, QC, Canada) for safe storage between experiments. All lambs were able to stand soon after the completion of surgery and were allowed to recover from the effects of surgery and anesthesia in a small animal critical care unit (Shor-line, Kansas City, KS, USA) for ∼30–60 min, after which time they were returned to the *vivarium*, where they were housed with their mothers until the time of the experiment at least 4 days later. Antibiotics (Excenal, ceftiofur 2 mg kg^−1^; Pfizer Canada Inc., Kirkland, QC, Canada) were administered intramuscularly at 24-h intervals beginning from the day before surgery for a total period of 4 days. During the recovery period, animals were removed from the *vivarium* for ∼1 h daily to allow them to accommodate to a supportive sling in which they were housed during experiments. This training period ensured that animals were at ease and resting quietly in the laboratory setting during experiments.

### Experimental details

On the day of an experiment, each animal was removed from the *vivarium* and placed in the same supportive sling in the laboratory environment for at least 60 min. During this equilibration period, an I.V. infusion of 5% dextrose in 0.9% sodium chloride (4.17 mL h kg^−1^) was started to assist in maintaining electrolyte balance throughout the experiment. The right femoral catheters were connected to pressure transducers (Statham, P23XL, West Warwick, RI, USA) for measuring arterial pressure. Arterial pressure was recorded onto a polygraph (Grass Instruments, model 7, West Warwick, RI, USA) and simultaneously digitized at 200 Hz using the data acquisition and analysis software package, PolyVIEW™ (Astro Med Inc., Grass Instrument Division, West Warwick, RI, USA). During experiments, body core temperature (Tc) was measured through a rectally inserted temperature probe (Thermalert TH-5, Physitemp, Clifton, NJ, USA).

Experiments consisted of cardiovascular measurements before and after hemorrhage of 30% of calculated vascular volume (hemorrhage, experiment one), or 0% of calculated vascular volume (sham hemorrhage, experiment two). The two experiments were carried out in random order at minimum intervals of 48 h using procedures similar to those previously detailed (Smith and Abu-Amarah 1997, 1998; Smith et al. 2000, 2004). Blood was removed at a constant rate over 10 min from the left femoral venous catheter and using a preprogrammable syringe pump (PHD 2000 Infusion/Withdrawal Dual Syringe Pump; Harvard Apparatus, Inc., Holliston, MA), based upon the recently standardizing protocol for severe hemorrhage in conscious rats by Ahlgren et al. (2007), and our previous assessments of vascular volume in conscious developing sheep aged ∼1 to ∼6 weeks of 75 mL kg^−1^ (Smith et al. 1997).

For each experiment, baseline cardiovascular variables were measured for a Control period of 30 min (C) at the end of which the arterial baroreflex control of heart rate was assessed (Sener and Smith 2001; Monument and Smith 2003; Qi and Smith 2007; Wehlage and Smith 2012): Briefly, phenylephrine hydrochloride (PHE, 10 *μ*g kg^−1^; Sabex Inc., Boucherville, QC, Canada), and sodium nitroprusside (SNP, 10 *μ*g kg^−1^; Sigma-Aldrich, Oakville, ON, Canada) were administered over 20 sec using a programmable syringe pump (Model 11; Harvard Apparatus) to increase and decrease arterial pressure from resting levels, and to allow the construction of the physiological range over which the arterial baroreflex operates including the upper and lower limits. Beat-to-beat systolic arterial pressure (SAP) and pulse interval were measured for 10 seconds before each intervention, and measurements continued until the maximum response was achieved. The arterial baroreflex was also assessed 30 min and 60 min after 0% (sham) and 30% hemorrhage.

At the end of the two experiments, lambs were administered a lethal dose of pentobarbitone, and upon *postmortem*, the positions of all catheters were verified.

### Data handling and analysis

Cardiovascular variables were averaged over 10 and 30-min periods using the analyses component of PolyVIEW.™ Heart rate was calculated using the peak intervals of the arterial pressure waveform. For each assessment of the arterial baroreflex control of heart rate, the relationship between SAP and heart rate obtained during the consecutive pulse interval was constructed for each animal. Data obtained from within an age group were pooled, and a four-parameter sigmoidal logistic function applied (SigmaPlot, version 11.0; Jandel Scientific, Richmond, CA, USA). The method used to assess the arterial baroreflex control of heart rate was that described by Kent et al. (1972) and detailed previously by us (Sener and Smith 2001; Monument and Smith 2003; Qi and Smith 2007; Wehlage and Smith 2012): heart rate = *P*_4_ + *P*_1_/[1 + *e*^*P*_2_ (SAP − *P*_3_)], where *P*_1_ is the heart rate range (beats min^−1^), *P*_2_ is the slope coefficient, *P*_3_ is SAP at the midpoint of heart rate range (mmHg), and *P*_4_ is the minimum heart rate (beats min^−1^), and maximum gain (*G*_*max*_) = −*P*_1_ × *P*_2_ × 0.25, and was considered to describe the sensitivity of the baroreflex control of heart rate [see also Jimbo et al. (1994)].

### Statistical analyses

For parameters governing the arterial baroreflex control of heart rate, statistical comparisons between group I and II lambs were made using Student’s nonpaired *t*-tests, and between Control, 30-min, and 60-min using Student’s paired *t*-tests with a Bonferroni correction. Other cardiovascular variables were assessed for statistical significance using ANOVA for repeated measures over time, factors being age (group I and II) and treatment (0% or sham hemorrhage and 30% hemorrhage) and a Student Newman–Keuls multiple comparison procedure where appropriate (using SPSS-PC version 16).

## Results

Baseline variables for groups I and II are provided in Table1. Heart rate was lower in group II as compared to group I lambs.

**Table 1:** Demographic and baseline measurements in conscious lambs

	Group I	Group II
*N*	7	7
Sex distribution	4♂, 3♀	6♂, 1♀
Body weight (kg)	8.6 ± 0.8	15.9 ± 1.2†
Age (days)	12 ± 2	43 ± 7†
Total kidney weight (g)	28.9 ± 7.8	46.3 ± 13.9†
Tc (°C)	39.7 ± 0.3	39.8 ± 0.4
MAP (mmHg)	74 ± 3	74 ± 6
SAP (mmHg)	107 ± 5	103 ± 8
DAP (mmHg)	53 ± 3	53 ± 6
HR (beats per min)	199 ± 24	124 ± 9†

Values are mean ± SD. Tc, core temperature; MAP, mean arterial pressure; SAP, systolic arterial pressure; DAP, diastolic arterial pressure; HR, heart rate.

† *P* < 0.05 compared to group I.

There was an overall effect of treatment on mean arterial pressure (*F* = 56.315, *P* < 0.001), systolic arterial pressure (*F* = 34.799, *P* < 0.001), and diastolic (*F* = 16.208, *P* < 0.001) but no age effects. Mean arterial pressure decreased by 10 min after hemorrhage and remained so for 60 min (Table2). There was an overall effect of age (*F* = 38.417, *P* < 0.001) and hemorrhage (*F* = 15.960, *P* < 0.001) on heart rate as well as a significant interaction between age and hemorrhage (*F* = 3.527, *P* < 0.05). In group I, heart rate decreased then returned toward control followed by an increase at 60 min. In contrast, heart rate remained constant after hemorrhage then increased (Table2).

**Table 2:** Cardiovascular responses to decompensated hemorrhage

	Group I			Group II		
	Control	30 min	60 min	Control	30 min	60 min
0% Hemorrhage						
MAP (mmHg)	75 ± 3	77 ± 5	76 ± 4	72 ± 6	72 ± 7	74 ± 6
SAP (mmHg)	109 ± 6	110 ± 5	112 ± 5	101 ± 8	100 ± 7	103 ± 8
DAP (mmHg)	54 ± 3	54 ± 6	52 ± 3	51 ± 5	52 ± 7	51 ± 4
HR (beats min^−1^)	197 ± 20	192 ± 18	195 ± 21	123 ± 5†	121 ± 3†	126 ± 18†
30% Hemorrhage						
MAP (mmHg)	73 ± 4	60 ± 10*	66 ± 6*	76 ± 5	58 ± 4*	68 ± 7*
SAP (mmHg)	105 ± 4	89 ± 9*	98 ± 11	105 ± 8	83 ± 5*	96 ± 8
DAP (mmHg)	52 ± 3	43 ± 11*	46 ± 5	54 ± 7	43 ± 4*	50 ± 8
HR (beats min^−1^)	200 ± 29	164 ± 24*	232 ± 45*	126 ± 12†	120 ± 21†	145 ± 17†

Values are mean ± SD; MAP, mean arterial pressure; SAP, systolic arterial pressure; DAP, diastolic arterial pressure and HR, heart rate.

* *P* < 0.05 compared to Control and

† *P* < 0.05 compared to group I.

With respect to parameters governing the arterial baroreflex control of heart rate, there was an overall effect of age (*F* = 49.92, *P* < 0.001) and treatment (*F* = 8.09, *P* < 0.05) on the heart rate range (*P1*). As illustrated in Figures4 and Table3, in both groups, by 30 min after 30% hemorrhage, there was a significant decrease in *P1*; by 60 min, values returned to C.

**Table 3:** Effects of decompensated hemorrhage on parameters governing the arterial baroreflex control of heart rate

	Group I			Group II		
	Control	30 min	60 min	Control	30 min	60 min
HR range, *P1* (beats min^−1^)	192 ± 13	102 ± 9*	190 ± 27	134 ± 21†	82 ± 10*	134 ± 15†
Slope coefficient, *P2*	0.1 ± 0.02	0.2 ± 0.1	0.2 ± 0.07	0.1 ± 0.04	0.2 ± 0.1	0.2 ± 0.1
SAP_50_*, P3* (mmHg)	98 ± 1	95 ± 2	106 ± 3*	107 ± 4†	97 ± 2	103 ± 2
Minimum HR, *P4* (beats min^−1^)	72 ± 10	59 ± 8	32 ± 25*	40 ± 15†	24 ± 9*†	20 ± 13*†
Maximum HR (beats min^−1^)	265 ± 12	168 ± 27*	226 ± 46*	167 ± 12†	103 ± 17*†	146 ± 20†
*G*_max_	5 ± 1	4 ± 2	6 ± 3	3 ± 1	3 ± 1	5 ± 3

Values are mean ± SD. SAP_50_, SAP at the midpoint of the curve; *G*_max_, maximum gain.

* *P* < 0.05 compared to Control and

† *P* < 0.05 compared to Group I.

**Figure 1:**
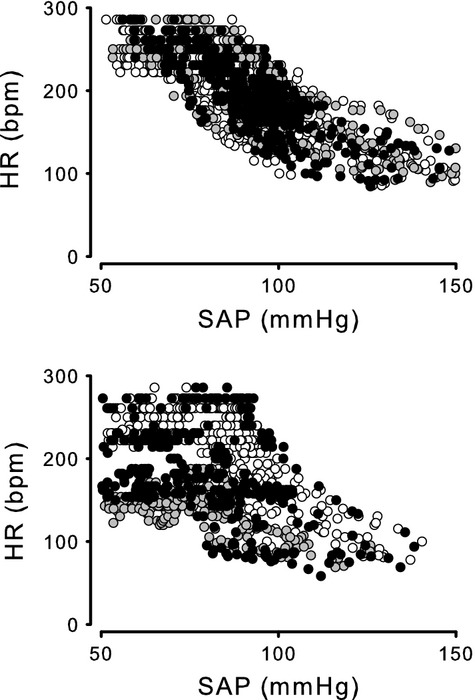
Effects of severe hemorrhage on the baroreflex in group I – raw data. Relationship between systolic arterial pressure (SAP) and heart rate (HR) measured in group I obtained before (Control, white symbols) and 30 min (grey symbols) and 60 min (black symbols) after 0% hemorrhage (top) and after 30% hemorrhage (bottom). Raw data were obtained during arterial baroreflex measurements in seven animals.

**Figure 2:**
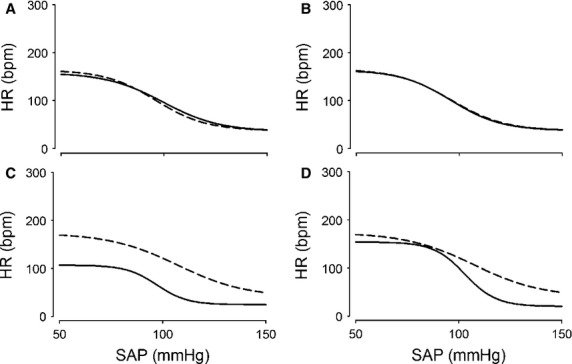
Effects of severe hemorrhage on the baroreflex in group I – average data. Relationship between systolic arterial pressure (SAP) and heart rate (HR) measured in group I obtained before (Control) and 30 min (A) and 60 min (B) after 0% hemorrhage and 30 min (C) and 60 min (D) after 30% hemorrhage. Four-parameter logistic function was applied to raw data presented in Figure1. Dotted line shows Control. Solid line shows responses to hemorrhage.

**Figure 3:**
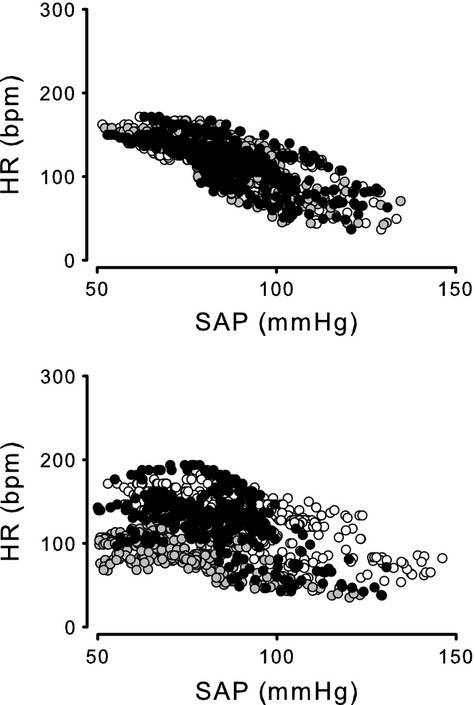
Effects of severe hemorrhage on the baroreflex in group II – raw data. Relationship between systolic arterial pressure (SAP) and heart rate (HR) measured in group II obtained before (Control, white symbols) and 30 min (grey symbols) and 60 min (black symbols) after 0% hemorrhage (top) and after 30% hemorrhage (bottom). Raw data were obtained during arterial baroreflex measurements in seven animals.

**Figure 4:**
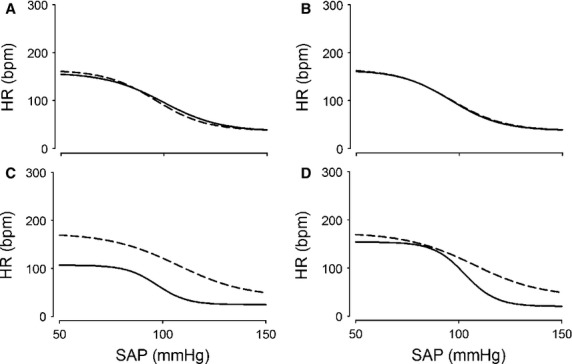
Effects of severe hemorrhage on the baroreflex in group II – averaged data. Relationship between systolic arterial pressure (SAP) and heart rate (HR) measured in group II before (Control) and 30 min (A) and 60 min (B) after 0% hemorrhage and 30 min (C) and 60 min (D) after 30% hemorrhage. Four-parameter logistic function was applied to raw data presented in Figure3. Dotted line shows Control. Solid line shows responses to hemorrhage.

**Figure 5:**
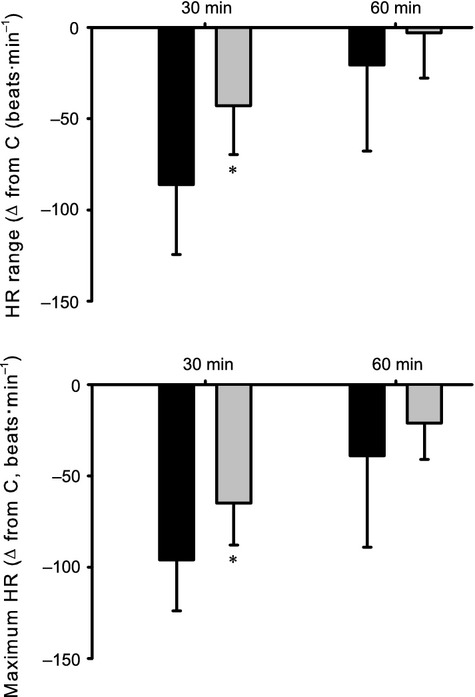
Effect of hemorrhage on the heart rate range, and maximum heart rate. Changes in heart rate range (top) and maximum heart rate (bottom) measured at 30 and 60 min compared to Control (C), after hemorrhage in both groups. *P < 0.05 compared to group I. Mean data are shown.

For the systolic arterial pressure at the midpoint of the heart rate range (*P3*), there was an effect of age (*F* = 6.38, *P* < 0.05) and treatment (*F* = 6.93, *P* < 0.05). As shown in Figures5, 60 min after 30% hemorrhage, there was an increase in *P3* in group I but not group II. Minimum heart rate (*P4*) decreased in both groups by 30 min after 30% hemorrhage; this response was greater at 30 min in group II as compared to group I, as reflected in an overall effect of age (*F* = 21.44, *P* < 0.001).

*G*_max_ was not significantly altered after hemorrhage in groups I and II (Table3). There were also no effects of sham hemorrhage on any of the measured or calculated variables including the parameters governing the arterial baroreflex control of heart rate in both groups and there was no evidence of any sex-related differences in the baroreflex responses to hemorrhage in either age group. Tc was not altered by sham hemorrhage or hemorrhage.

## Discussion

The aim of the present study was to investigate in the newborn period and as postnatal maturation proceeds, hemodynamic responses to severe hemorrhage beyond the critical point and at which compensatory mechanisms fail. To this end, cardiovascular variables and the arterial baroreflex control of heart rate were assessed before and after hemorrhage of 30% as well as sham hemorrhage at two stages of postnatal maturation in conscious lambs. Novel findings of our study are as follows: (1) Within 30 min of severe hemorrhage, both age groups sustained a decrease in mean arterial pressure which resulted from changes in both systolic and diastolic blood pressures; (2) there was a decrease in heart rate in group I but not group II at 30 min followed by an increase at 60 min in both groups; (3) both *P1* and *P4* decreased in both groups by 30 min; (4) *P1* returned to Control by 60 min, whereas *P4* remained decreased; (5) sham hemorrhage elicited no physiological responses in either age group. These novel observations include the first assessment of the arterial baroreflex before and after hemorrhage in conscious newborn animals, and reveal age-dependent effects of severe degrees of blood loss on several parameters governing the arterial baroreflex early in life.

Little evidence exists regarding hemorrhagic shock early in life. This is surprising because hypovolemia and hypovolemic shock are central entities in the pattern of morbidity and mortality and include blood loss before birth as well as at the time of delivery. In previous studies in conscious sheep, we reported some of the first comprehensive reports of the physiological responses to compensated hemorrhage in the newborn period (Smith and Abu-Amarah 1997, 1998; Smith et al. 2000, 2004; Smith 2002). These investigations revealed that the newborn appears to recruit different mechanisms to restore blood pressure following compensated blood loss as compared to later in life. The present findings show that age also influences the responses to more severe blood loss: both age groups failed to compensate rapidly following blood loss, since blood pressure remained decreased 30 min after hemorrhage and in the absence of intervention; at this time, there was no evidence of compensation. There was, however, evidence of partial compensation occurring by one hour after blood loss suggesting that by this time, additional mechanisms had been recruited to assist in the return of blood pressure toward control in both age groups.

The profile of the heart rate response was, however, quite different in newborns as compared to older animals. Studies in adult humans have provided evidence that hypovolemic shock with tachycardia may represent a transition to an irreversible stage. For example Secher et al. (1992) divided the heart rate response to hypovolemia into three stages. First, a modest increase in heart rate occurs and blood pressure is maintained with moderate blood loss; second, a reduction in blood volume by ∼30% leads to a decrease in heart rate as well as blood pressure; in the third stage, blood pressure falls further as blood loss continues and a tachycardia of >120 beats per min is observed. This latter stage results in irreversible shock with death if untreated. In the present study in conscious lambs, a tachycardia was seen in the youngest group by 60 min after hemorrhage and after an initial decrease in heart rate, whereas in the older animals, there were no significant changes in heart rate after severe blood loss. This might suggest that the newborn animal was in a more precarious cardiovascular situation from a clinical perspective as compared to the older animals. Indeed, this younger age group might have proceeded rapidly to a state of irreversible shock if no intervention (i.e., return of blood) occurred.

Alterations in the baroreflex following blood loss were also different in the two age groups of animals, demonstrating a developmentally regulated baroreflex response to hemorrhage. The changes appeared to be more dramatic in the youngest age group with a marked decrease in the operating range and maximum heart rate at 30 min. This indicates that there was a limit to the baroreflex control of heart rate following severe blood loss by 30 min. In older animals, there was also a decrease in the operating range and in the maximum heart rate after blood loss but this response was considerably less than that which occurred in the younger age group. As well, the return of the baroreflex toward control was achieved more quickly in the older animals with the maximum heart rate reached by 60 min. In younger animals, maximum heart rate remained below control levels. The mechanisms underlying both the baroreflex responses to blood loss, and the age-dependent differences, are not known. Moreover, there have been no studies for comparison purposes in which the entire or complete baroreflex has been directly assessed before, during and after hemorrhage at any age.

In previous studies, we explored some of the mechanisms that regulate the arterial baroreflex control of heart rate in the newborn period (Sener and Smith 2001; Monument and Smith 2003; Qi and Smith 2007; Wehlage and Smith 2012). Our research findings demonstrate that angiotensin II (Ang II) is one of the most important vasoactive regulatory peptides. For example, endogenously produced Ang II regulates resting heart rate and blood pressure as well as the baroreflex, through activation of type 1 receptors, or AT1Rs (Wehlage and Smith 2012). Alterations in the arterial baroreflex in response to severe hemorrhage in the present study may, therefore, have resulted from activation of the renin–angiotensin system. Our previous studies into compensated hemorrhage have shown that the renin–angiotensin system is activated by as little as 10–15% of vascular volume, with effects being greater in newborn animals as compared to young sheep (Smith et al. 2000). It is, however, unlikely that this is the primary factor since it appears that the renin–angiotensin system does not contribute to cardiovascular responses to such a severe level of blood loss [see review by Schadt and Ludbrook (1991)].

We also demonstrated that activation of kappa opioid receptors (KORs) has a profound effect on the baroreflex in conscious lambs (Qi and Smith 2007). We speculate, therefore, that the shift in the baroreflex after hemorrhage in the present study may have resulted from altered activity of KORs within the central nervous system (CNS). For example, it is known that microinjection of either a KOR agonist, or dynorphin, the naturally occurring ligand for KORs, into the anterior hypothalamus in the rat (Fan et al. 1993) decreases blood pressure and attenuates cardiovascular recovery following hemorrhage suggesting that activation of KORs attenuates cardiovascular responses to blood loss. In addition, Feuerstein et al. (1985) measured a 50% reduction in immunoreactive dynorphin A in pituitary and brain nuclei of conscious rats at 24 h after 40% hemorrhage. In anaesthetised rats, KORs are involved in decompensated hemorrhage at the level of the caudal midline medulla (Henderson et al. 2002) and activation of KORs lowers blood pressure and inhibits recovery from blood loss. In conscious adult sheep, activation of KORs in hemorrhagic shock also initiates hypotension and bradycardia (Frithiof and Rundgren 2006; Frithiof et al. 2007) supporting the earlier observations in anesthetised rats that dynorphin content is depleted in several sites within the CNS following decompensated hemorrhage (Feuerstein et al. 1984, 1985). An inhibitory effect of a KOR agonist on blood pressure recovery from hypovolemia, also suggests a central cardiodepressor action of dynorphins. Furthermore, microinjection of dynorphins into specific sites within the hypothalamus worsens cardiovascular recovery from hypovolemia (Fan and McIntosh 1994). Further investigations are clearly warranted to determine the role of KORs in the cardiovascular responses to severe hemorrhage early in life.

Ahlgren et al. (2007) carried out experiments focused on standardizing a protocol for severe hemorrhage in conscious rats. Their results suggested that a constant withdrawal rate until 30% of total blood volume had been removed produced the most reliable pattern of tachycardia and compensation initially, followed by hypotension and bradycardia or decompensation. In keeping with this observation in conscious rats, we conducted preliminary studies in conscious lambs aged ∼1 and ∼ 6 weeks and determined the “critical point” – beyond which compensatory responses to blood loss fail. These preliminary experiments revealed a lack of compensation after a blood loss of ∼24 to 26% of vascular volume following a constant withdrawal rate, such that blood pressure remained decreased after 30 min and in the absence of intervention. Therefore, in the current experiments, 30% of blood volume was removed at a constant rate and standardized according to vascular volume, to allow for comparisons across the two age groups. A fixed volume hemorrhage as used in the present experiments allows the study of intrinsic hemodynamic responses that would occur in the state of hemorrhagic shock and in the absence of clinical intervention.

In the present experiments, by 60 min after blood loss, there was evidence of partial recovery in some of the measured variables suggesting that partial compensatory responses were occurring. For example, SAP and diastolic arterial pressure (DAP) were returning toward control levels although mean arterial pressure (MAP) remained decreased. Therefore, our data show that the decompensatory response present by 30 min after blood loss is not sustained. This could be the result of alterations in endocrine responses to blood loss (e.g., dynorphins), or decreased renal function, since the response was slowly occurring. Further studies are warranted to evaluate the possible mechanisms underlying the responses to blood loss that we have described herein for the developing conscious lamb.

In conclusion, the results of the present research provide new information that cardiovascular responses to severe hemorrhage are age dependent. The mechanism(s) underlying these responses have not been determined but could be related to opioid receptors (specifically kappa opioid peptides) or other factors that are known to modulate the arterial baroreflex. Future studies will be important to investigate the underlying mechanisms of these age-dependent responses to hemorrhage.
