# Erratum

**DOI:** 10.14814/phy2.12585

**Published:** 2015-10-05

In [1], the incorrect Figure 2 was published on page 5.

Below is the correct Figure 2.


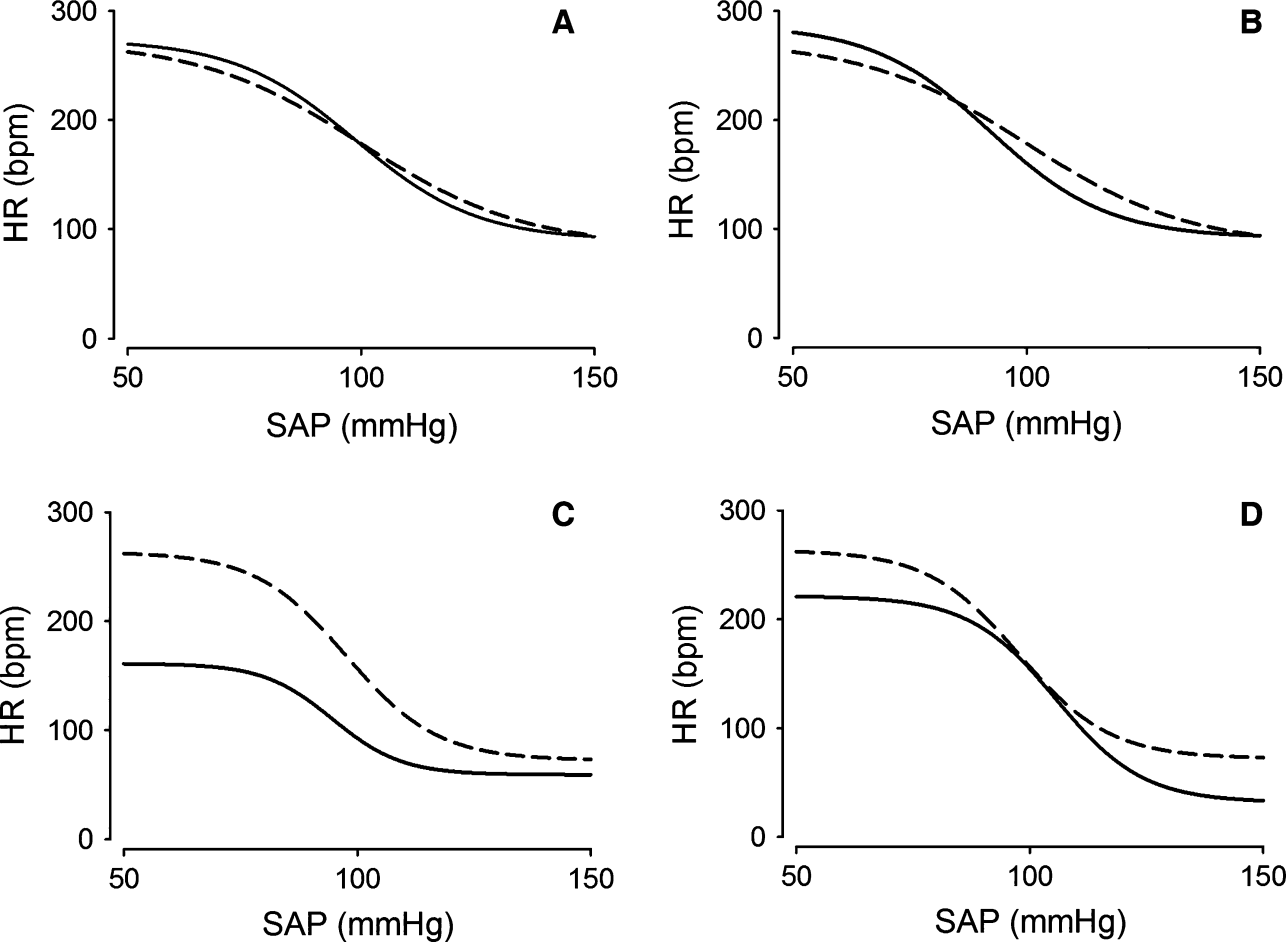


The authors apologize for this error.
